# Association between oral corticosteroid use and pyogenic liver abscesses in a case-control study

**DOI:** 10.1051/bmdcn/2018080105

**Published:** 2018-02-26

**Authors:** Shih-Wei Lai, Cheng-Li Lin, Kuan-Fu Liao

**Affiliations:** 1 College of Medicine, China Medical University Taichung 404 Taiwan; 2 Department of Family Medicine, China Medical University Hospital Taichung 404 Taiwan; 3 Management Office for Health Data, China Medical University Hospital Taichung 404 Taiwan; 4 College of Medicine, Tzu Chi University Hualien 970 Taiwan; 5 Department of Internal Medicine, Taichung Tzu Chi General Hospital Taichung 427 Taiwan

**Keywords:** Oral corticosteroids, Pyogenic liver abscesses, Case-control study, Taiwan

## Abstract

Background and aim: There are no epidemiological studies focusing on the association between oral corticosteroid use and pyogenic liver abscesses. The aim of the study was to assess whether oral corticosteroid use is associated with increased odds of pyogenic liver abscesses in adults in Taiwan.

Methods: This retrospective population-based case-control study was conducted to analyze the database of the Taiwan National Health Insurance Program from 2000 to 2013. Subjects aged 20 to 84 years with their first episode of pyogenic liver abscesses were assigned as the cases (n = 881). Randomly selected subjects without pyogenic liver abscesses aged 20 to 84 years were selected as the controls (n = 3207). A multivariable logistic regression model was used to assess the odds ratio and 95% confidence interval for the correlation of oral corticosteroid use with pyogenic liver abscesses.

Results: After regulating for confounders, the adjusted odds ratio of pyogenic liver abscesses was 1.40 for subjects currently using oral corticosteroids (95% confidence interval 1.14, 1.70), compared with subjects who never used them. Upon further analysis, the adjusted odds ratio of pyogenic liver abscesses was 1.03 for subjects with current use of oral corticosteroids when increasing dosage for every one mg (95% CI 1.01, 1.06).

Conclusion: Although the findings are not unexpected, they are important because they suggest that current use of oral corticosteroids is significantly associated with increased odds of developing pyogenic liver abscesses in adults in Taiwan, with a dose-dependent effect.

## 1. Introduction

Clinically, a pyogenic liver abscess can be a serious disease due to its potentially high mortality rate. Previous studies have shown that its mortality rate is between 5.6% to 11.7%, [1, 2] depending on the population studied. Current evidence has shown that a wide range of underlying conditions could be associated with having pyogenic liver abscesses, conditions including biliary disease, diabetes mellitus, malignancy, having a splenectomy or appendectomy, and herpes zoster, [1-5] but corticosteroid use’s risk for developing pyogenic liver abscesses has not yet been studied.

Previous studies have shown that corticosteroid use is associated with an increased risk of life-threatening infectious events such as tuberculosis, herpes zoster, pneumonia, urinary tract infection, gastroenteritis, and postoperative infection, all of which are due to the anti-inflammatory and immunosuppressive effects associated with corticosteroid use. [6-9] Based on the above clinical findings, we hypothesized that corticosteroid use may also be associated with an increased risk of developing pyogenic liver abscesses. To date, the association between corticosteroids use and pyogenic liver abscess has not yet been reported. If pyogenic liver abscesses are associated with corticosteroid use, physicians should be aware of the possible risk of pyogenic liver abscesses in patients using corticosteroids. Therefore, we conducted a retrospective population-based case-control study to analyze the Taiwan National Health Insurance (NHI) Program database to investigate whether (1) corticosteroid use is associated with increased odds of developing pyogenic liver abscesses; and (2) the dosage of corticosteroid use is associated with increased odds of developing pyogenic liver abscesses.

## 2. Methods

### 2.1. Study design and data source

The methodology used in this study was adapted from previous studies. [10-12] It is not necessary to repeat published protocol details. Thus, we have summarized it as follows and cited the relevant sources. Taiwan is an independent country with more than 23 million residents. [13-30] We designed a populationbased case-control study to analyze the database of the Taiwan National Health Insurance Program. The program was launched in March 1995, and it now covers almost 99% of the residents living in Taiwan. [31] Briefly, all diseases included in the database are diagnosed according to the International Classification of Diseases, Ninth Revision, Clinical Modification (ICD-9 codes). The database includes sex and date of birth, and other data such as outpatient care, inpatient care, dental care, emergency care, and prescription medications for everyone enrolled. Personal identification has been scrambled to maintain confidentiality. The database is open for public access. The program has been described in detail previously. [32-36] As an addendum, the Research Ethics Committee of China Medical University and Hospital in Taiwan approved this study (CMUH-104-REC2-115).

### 2.2. Study subjects

**Fig. 1:**
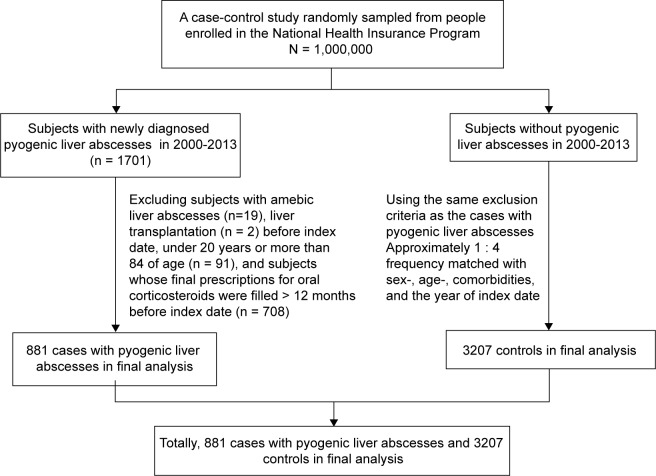
Flow chart showing the selection of cases with pyogenic liver abscesses and controls for the study.

We selected subjects aged 20 to 84 years with their first episode of pyogenic liver abscesses (ICD-9 code 572.0) from 2000 to 2013 as the cases with pyogenic liver abscesses. The index date was defined as the date of cases being diagnosed with their first episode of pyogenic liver abscesses. We selected subjects without a diagnosis of a pyogenic liver abscess aged 20 to 84 years from the same database as the controls. Subjects who had a prior diagnosis of amebic liver abscess or liver transplantation before the index date were excluded from the study. The cases and the controls were matched by sex, age (every 5-year interval), comorbidities, and the year of their index date (Fig. 1).

### 2.3. Potential confounders

There are other known risk factors for pyogenic liver abscesses which should be assessed as potential confounders, including alcohol-related diseases, biliary stones, chronic kidney diseases, diabetes mellitus, as well as chronic liver diseases including cirrhosis, hepatitis B, hepatitis C, and other chronic hepatitis. All comorbidities were diagnosed based on ICD-9 codes. The accuracy of ICD-9 codes has been extensively discussed in previous studies. [37-49]

### 2.4. Definition of oral corticosteroid exposure

It is hard to measure the dosage for topical corticosteroids or inhaled corticosteroids. Long-term use of injected corticosteroids is also rarely found at outpatient department. Therefore, only oral corticosteroids were included for detailed analysis. For our purposes, topical, inhaled, and injected corticosteroids were combined as other forms of corticosteroids. Oral corticosteroids available in Taiwan are as follows: cortisone, dexamethasone, fludrocortisone, methylprednisolone, prednisolone, and triamcinolone. To assess the correlation of oral corticosteroid use with pyogenic liver abscesses, the prescription history of oral corticosteroids before the index date. In order to reduce biased results, subjects whose final prescriptions for oral corticosteroids were filled > 12 months before the index date were excluded from the study. Therefore, only subjects whose final prescriptions for oral corticosteroids were filled . 12 months before the index date were included. In Taiwan, prescriptions for chronic diseases are refilled every 3 months. Therefore, oral corticosteroid use was categorized in this study according to the final prescription for oral corticosteroids being filled within 3 months, between 3-6 months, and between 6-12 months before the index date [current use (< 3 months), recent use (3-6 months), and past use (6-12 months)], a structure which was adapted from previous studies. [50-52] Subjects who never had a prescription for oral corticosteroids were defined as never used.

### 2.5. Statistical analysis

Distributions of sex, age, oral corticosteroid use, other forms of corticosteroids, and comorbidities between the cases and the controls were analyzed by using a *Chi*-square test for categorized variables and a *t*-test for continuous variables. In the beginning, all variables were included in a univariable logistic regression model. Only variables found to be statistically significant in the univariable model were further examined in a multivariable logistic regression model. The odds ratio (OR) and 95% confidence interval (CI) were used to assess the correlation of oral corticosteroid use with pyogenic liver abscesses. We further conducted an analysis on the dose-dependent assessment among subjects in current use of oral corticosteroids category. All data processing and statistical analyses were performed with the SAS software version 9.2 (SAS Institute, Inc., Cary, North Carolina, USA). A two-tailed *P* value < 0.05 was considered statistically significant.

## 3. Results

### 3.1. Characteristics of the study population

Table 1 shows the distributions of sex, age, oral corticosteroid use, other forms of corticosteroids, and comorbidities between the cases and controls. The study included 881 cases with pyogenic liver abscesses and 3207 controls, with similar distributions of sex and age. The mean ages (standard deviation) of the study subjects were 59.7 (14.1) for the cases and 59.5 (14.0) for the controls (t-test, *P* = 0.72). The cases had a statistically higher proportion of current use of oral corticosteroids than the controls (20.1% vs. 14.8%, *Chi*-square test, *P* = 0.002). The cases also had statistically higher proportions of alcohol-related diseases, biliary stones, and chronic kidney diseases than the controls (*Chi*-square test, *P* < 0.05).

**Table 1:** Clinical characteristics of cases with pyogenic liver abscess and sex- and age-matched controls.

	Cases N = 881		Controls N = 3207		
			
Variable	n	(%)	n	(%)	P value*
Sex					0.75
Female	301	(34.2)	1077	(33.6)	
Male	580	(65.8)	2130	(66.4)	
Age group (years)					0.98
20-39	81	(9.2)	291	(9.1)	
40-64	460	(52.2)	1688	(52.6)	
65-84	340	(38.6)	1228	(38.3)	
Age (years), mean ± standard deviation†	59.7	± 14.1	59.5 ±	14.0	0.72
Oral corticosteroid use					0.002
Never used	539	(61.2)	2121	(66.1)	
Current use	177	(20.1)	473	(14.8)	
Recent use	63	(7.1)	235	(7.3)	
Past use	102	(11.6)	378	(11.8)	
Ever used other forms of corticosteroids	344	(39.1)	1314	(41.0)	0.30
Comorbidities					
Alcohol-related diseases	65	(7.38)	169	(5.27)	0.02
Biliary stones	113	(12.8)	276	(8.61)	<0.001
Chronic kidney diseases	56	(6.36)	138	(4.30)	0.01
Chronic liver diseases	235	(26.7)	774	(24.1)	0.12
Diabetes mellitus	252	(28.6)	816	(25.4)	0.06

Data are presented as the number of subjects in each group with percentages given in parentheses.

* *Chi*-square test, and

† *t*-test comparing subjects with and without pyogenic liver abscess.

### 3.2. Correlation of oral corticosteroid use with pyogenic liver abscesses

Variables found to be statistically significant in the univariable model were further examined in the multivariable model. After adjusting for alcohol-related diseases, biliary stones, and chronic kidney diseases, the multivariable logistic regression model showed that the adjusted OR of pyogenic liver abscesses was 1.40 for subjects in the current use of oral corticosteroids category (95% confidence interval 1.14, 1.70), compared with the never use category. The adjusted ORs decreased to 1.01 for those in the recent use of oral corticosteroids category (95% CI 0.76, 1.36), and 1.03 for those in the past use of oral corticosteroids category (95% CI 0.81, 1.31), but there was no statistical significance (Table 2).

**Table 2:** Odds ratio and 95% confidence interval of pyogenic liver abscesses associated with oral orticosteroid use, other forms of corticosteroids, and comorbidities.

	Crude		Adjusted †	
		
Variable	OR	(95% CI)	OR	(95% CI)
Sex (male *vs.* female)	0.97	(0.83, 1.14)		
Age (per year)	1.00	(0.99, 1.01)		
Oral corticosteroid use (never used as a reference)				
Current use	1.47	(1.21, 1.79)	1.40	(1.14, 1.70)
Recent use	1.06	(0.79, 1.42)	1.01	(0.76, 1.36)
Past use	1.06	(0.84, 1.35)	1.03	(0.81, 1.31)
Other forms of corticosteroids (never used as a reference)				
Ever used	0.92	(0.79, 1.08)		
Comorbidities (yes *vs*. no)				
Alcohol-related diseases	1.43	(1.07, 1.93)	1.47	(1.09, 1.98)
Biliary stones	1.56	(1.24, 1.97)	1.59	(1.26, 2.01)
Chronic kidney diseases	1.51	(1.10, 2.08)	1.53	(1.10, 2.11)
Chronic liver diseases	1.14	(0.97, 1.36)		
Diabetes mellitus	1.17	(0.99, 1.39)		

† Variables found to be statistically significant in a univariable model were further examined in a multivariable model. Adjusted for alcohol-related diseases, biliary stones, and chronic kidney diseases.

### 3.3. Correlation of cumulative dosage of current use of oral corticosteroid with pyogenic liver abscesses

We further conducted an analysis on the dose-dependent assessment among subjects in the current use of oral corticosteroids category. After adjusting for alcohol-related diseases, biliary stones, and chronic kidney diseases, the adjusted OR of pyogenic liver abscesses was 1.03 for subjects in the current use of oral corticosteroids category when increasing dosage for every one mg (95% CI 1.03, 1.06), compared with the never used category (Table 3). There seems to be a dose-dependent effect of oral corticosteroid on the risk of developing pyogenic liver abscesses.

**Table 3:** Risk of developing pyogenic liver abscesses associated with cumulative dosage of current use of oral corticosteroids category.

Variable	Case number / Control number	Crude OR	(95% CI)	Adjusted OR†	(95% CI)
Never used corticosteroids as a reference	531/2121	1.00	(reference)	1.00	(reference)
Currently use oral corticosteroids (increase in dosage for every one mg)	350/1086	1.03	(1.01, 1.06)	1.03	(1.01, 1.06)

† Variables found to be statistically significant in a univariable model were further examined in a multivariable model. Adjusted for alcohol-related diseases, biliary stones, and chronic kidney diseases.

### 3.4. Interaction of effects between current use of oral corticosteroids and comorbidities on risk of developing pyogenic liver abscesses

Table 4 shows the risk of developing pyogenic liver abscesses stratified by current use of oral corticosteroids and comorbidities. When compared with subjects in the never used category of oral corticosteroids and without comorbidities including alcoholrelated diseases, biliary stones, and chronic kidney diseases, the odds ratio of having pyogenic liver abscesses was 1.6 among subjects in the current use of oral corticosteroids category alone and without comorbidities (95% CI 1.27, 2.01).

**Table 4:** Interaction of effects between current use of oral corticosteroids and comorbidities on the risk of developing pyogenic liver abscesses.

Variable			OR	(95% CI)
Oral corticosteroids	Comorbidities*	Case number/ control number		
Never used	No	423/1802	1.00	(Reference)
Never used	Yes	116/319	1.55	(1.22, 1.96)
Current use	No	127/339	1.60	(1.27, 2.01)
Current use	Yes	50/134	1.59	(1.13, 2.24)

* Comorbidities include alcohol-related diseases, biliary stones, and chronic kidney diseases.

## 4. Discussion

Because it is not unexpected that oral corticosteroid use would be associated with an increased risk of pyogenic liver abscesses, little research has focused on the association between oral corticosteroids use and pyogenic liver abscesses. Therefore, we cannot compare them with each other. Because there was no statistical difference with the other forms of corticosteroids between the cases and controls (Table 1), the confounding effects of other forms of corticosteroids impacting on the risk of developing pyogenic liver abscesses can be minimized. The present study showed that current use of oral corticosteroids is significantly associated with 1.4-fold increased odds of developing pyogenic liver abscesses, while recent use and past use did not show statistical significances. These findings are partially consistent with Sadr-Azodi *et al.*’s study reporting that only current use of oral corticosteroids was significantly associated with increased odds of developing acute pancreatitis (adjusted OR 1.53, 95% CI 1.27, 1.84), while recent users and past users did not have an increased risk of developing acute pancreatitis. [50] The above findings highlight that only subjects with a persistent use of oral corticosteroids would have the risk of developing pyogenic liver abscesses. Subjects who discontinue use of oral corticosteroids would not have this risk of developing pyogenic liver abscesses.

Upon further analysis, we found that the adjusted odds ratio is quantitatively small, but subjects in the current use of oral corticosteroids category had higher odds when increasing dosage for every one mg (adjusted OR 1.03, Table 3). This finding indicates that there seems to be a dose-dependent effect of oral corticosteroids on the risk of developing pyogenic liver abscesses. That is, the higher the dose of oral corticosteroids, the greater the risk of pyogenic liver abscesses.

Although both cases and controls were matched with comorbidities, the cases still had significantly higher proportions of alcohol-related diseases, biliary stones, chronic kidney diseases, and diabetes mellitus than the controls. There might be a bias in that patients with higher degrees of comorbidity were more likely to have been prescribed for oral corticosteroids. To reduce the confounding effects of comorbidities, we made a stratified analysis. We found that subjects in the current use of oral corticosteroids category and without comorbidities were associated with 1.6-fold increased odds of developing pyogenic liver abscesses, compared with subjects in the never used category of oral corticosteroids who did not have comorbidities (Table 4). This finding indicates that even without comorbidities, current use of oral corticosteroids alone still has an independent effect on the risk of developing pyogenic liver abscesses.

The real pathogenesis underlying the correlation of oral corticosteroid use and pyogenic liver abscesses cannot be absolutely studied in an observational study. However, previous studies have shown that corticosteroids can inhibit lymphocytes binding to endothelium *in vitro*, which further causes a down-regulation of immune functions. [53] In addition, corticosteroids can induce dose-dependent lymphocytopenia and can inhibit mononuclear cells releasing cytokine in a human study. [54] These effects result in an immunosuppressive status, which further increases the potential risk of infections such as tuberculosis, herpes zoster, pneumonia, urinary tract infection, gastroenteritis, and postoperative infections. [6-9] Our findings also highlight that, in addition to the above infections, oral corticosteroids use is associated with greater odds of developing pyogenic liver abscesses, also showing a dose-dependent effect.

The present study does have some limitations. First, due to the natural limitations of a retrospective study, the indication for oral corticosteroid therapy was not available, but it is essential to understand the relative risk-benefit of this intervention. Confounding with this undefined indication could contribute to the risk of developing pyogenic liver abscesses. This circumstance indicates a future research direction. Second, given that an immortal time bias is not easily overcome in a retrospective cohort design (users of oral corticosteroids vs. non-users of oral corticosteroids) that most studies have used to assess the association between oral corticosteroids and chronic conditions, that is why we used a case-control design (pyogenic liver abscess vs. no pyogenic liver abscess) to reduce immortal time bias. However, a cause and effect relationship cannot be determined in a case-control design. Third, because the eligible case number was rather small, we could not analyze the dosage of individual oral corticosteroids associated with the risk of developing pyogenic liver abscesses. The present study only showed the odds of developing pyogenic liver abscesses as associated with current use of overall oral corticosteroids (Table 2). A further study with a larger case number is suggested to analyze the dosage of individual oral corticosteroids associated with developing pyogenic liver abscesses.

Despite the above limitations, some strengths should be mentioned. Although the findings are not unexpected, no confirmatory research has been reported on the topic until now. To the best of our knowledge, this is the first epidemiological study to assess the correlation of oral corticosteroid use with pyogenic liver abscesses. It adds to the substantial amount of evidence and knowledge. We used a national database unique to Taiwan to conduct a well constructed and informative analysis, which is one of the inherent strengths of the present study. Several potential confounders were included for adjustment to reduce bias. The results are thus sound and straight-forward. The study’s conclusions are justified by its data and analysis. The findings may be of interest to researchers and clinicians interested in pyogenic liver abscesses.

We conclude that the adjusted odds ratio is quantitatively small, but current use of oral corticosteroids is significantly associated with greater odds of developing pyogenic liver abscesses in adults in Taiwan. There is a dose-dependent effect of oral corticosteroids on the risk of developing pyogenic liver abscesses. Even in the absence of comorbidities, current use of oral corticosteroids alone still has a unique effect on the risk of developing pyogenic liver abscesses. Physicians should be aware of the possible risk of developing pyogenic liver abscesses in patients using oral corticosteroids.

## Specific author contributions

Shih-Wei Lai contributed to the conception of the article, initiated the draft of the article, and revised the article. Cheng-Li Lin conducted data analysis and revised the article. Kuan-Fu Liao participated in data interpretation and revised the article.

## Conflict of Interest Statement

The authors disclose no conflicts of interest.
